# Pore forming channels as a drug delivery system for photodynamic therapy in cancer associated with nanoscintillators

**DOI:** 10.18632/oncotarget.25150

**Published:** 2018-05-18

**Authors:** Luiz Anastacio Alves, Leonardo Braga Ferreira, Paulo Furtado Pacheco, Edith Alejandra Carreño Mendivelso, Pedro Celso Nogueira Teixeira, Robson Xavier Faria

**Affiliations:** ^1^ Laboratório de Comunicação Celular, Instituto Oswaldo Cruz, Fundação Oswaldo Cruz-FIOCRUZ, 21045-900, Rio de Janeiro, RJ, Brasil; ^2^ Laboratório de Inflamação e Instituto Oswaldo Cruz, Fundação Oswaldo Cruz-FIOCRUZ, 21045-900, Rio de Janeiro, RJ, Brasil; ^3^ Laboratório de Toxoplasmose Instituto Oswaldo Cruz, Fundação Oswaldo Cruz-FIOCRUZ, 21045-900, Rio de Janeiro, RJ, Brasil

**Keywords:** drug delivery, cancer, PDT, nanoscintillators, pore forming channels

## Abstract

According to the World Health Organization (WHO), cancer is one of main causes of death worldwide, with 8.2 million people dying from this disease in 2012. Because of this, new forms of treatments or improvement of current treatments are crucial. In this regard, Photodynamic therapy (PDT) has been used to successfully treat cancers that can be easily accessed externally or by fibre-optic endoscopes, such as skin, bladder and esophagus cancers. In addition, this therapy can used alongside radiotherapy and chemotherapy in order to kill cancer cells. The main problem in implementing PDT is penetration of visible light deeper than 10 mm in tissues, due to scattering and absorption by tissue chromophores. Unfortunately, this excludes several internal organs affected by cancer. Another issue in this regard is the use of a selective cancer cell-photosensitizing compound. Nevertheless, several groups have recently developed scintillation nanoparticles, which can be stimulated by X-rays, thereby making this a possible solution for light production in deeper tissues. Alternative approaches have also been developed, such as photosensitizer structure modifications and cell membrane permeabilizing agents. In this context, certain channels lead to transitory plasma membrane permeability changes, such as pannexin, connexin hemmichannels, TRPV1-4 and P2×7, which allow for the non-selective passage of molecules up to 1,000 Da. Herein, we discuss the particular case of the P2×7 receptor-associated pore as a drug delivery system for hydrophilic substances to be applied in PDT, which could also be carried out with other channels. Methylene blue (MB) is a low cost dye used as a prototype photosensitizer, approved for clinical use in several other clinical conditions, as well as photodynamic therapy for fungi infections.

## INTRODUCTION

### Photodynamic action: a brief history of cancer treatment

In the western World, the term photodynamic action, a literal translation of the German word “Wirkung der photodynamischen”, was coined by von Tappeiner and collaborators to differentiate photodynamic action from the sensitization that occurs in photography films [1, 2]. At the time, Von Tappeiner's group was investigating the effect of antimalarial drugs on certain protozoa. A PhD student, Raab, discovered that certain dyes, such as Acridine, display phototoxic effects on *Paramaecium caudatum* only when exposed to sunlight [3, 4]. This was observed by chance, with controversial results, since the effects apparently depended on the sunlight conditions of their Laboratory at the Munich University. It is relevant to point out that Marcacci had recognized the phototoxicity of quinine on oocytes [5], but this seems to have been unknown by Von Tappeiner's team at the time.

Von Tappeiner quickly recognized the clinical potential of Raab's data, since he was apparently familiar with the work of a French neurologist that attempted to apply eosin to treat epilepsy, causing photoxicity skin effects [6]. Possibly based on that study, and on their own data, Von Tappeiner, H., and Jesionek, A. treated three patients presenting skin cancer and other pathologies with Photodynamic Therapy (PDT) [7]. In 1905, they treated more five patients presenting skin cancer, with good results [8]. The use of PDT to treat *in vivo* tumors was little explored until 1972, when Diamond and co-workers demonstrated a small decrease in tumors derived from a glioma cell line implanted in rats exposed to light and treated with haematoporphyrin [9]. In 1975, a breakthrough study was published on eleven human bladder carcinomas xenografted to artificially immunosuppressed mice treated with PDT, that demonstrated remarkable damage to the tumors [10]. Since then, PDT has been used to treat several cancer types easily exposed to light sources [11]. Most are skin cancers or tumors easily accessed by endoscopy devices. The largest obstacle for PDT application is the light necessary to reach deep into the cancer tissues.

### General photodynamic therapy aspects

**Figure 1:**
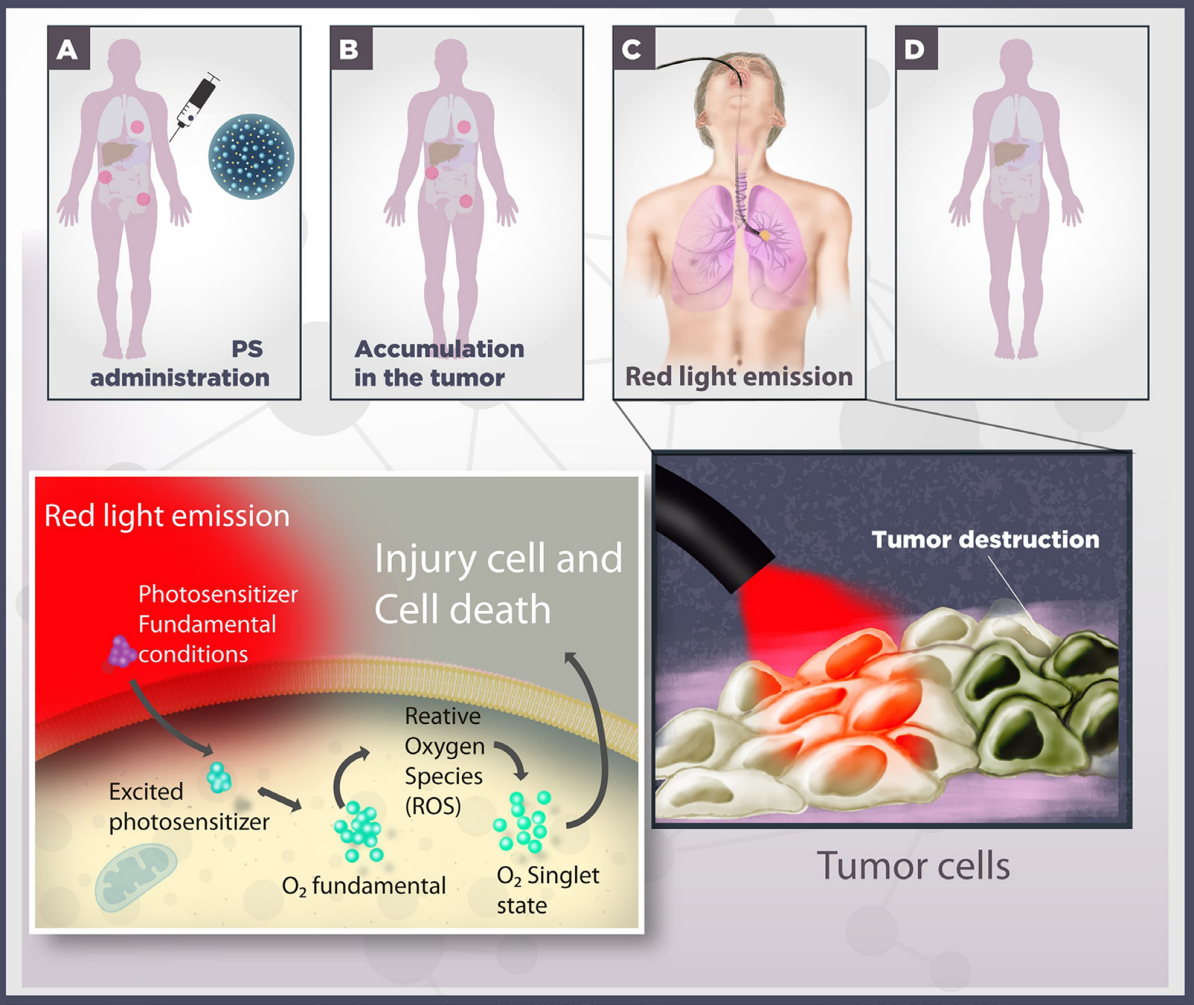
Schematic representation of photodynamic therapy (**A**) A photosensitizer (PS) is systemically or topically administered. (**B**) After systemic PS distribution, it selectively accumulates in the tumor, represented by red circles. (**C**) In cooperation to laparoscopic techniques, the cancer cells are irradiated with red light. Irradiation activates the PS and triggers a photochemical reaction in the presence of molecular oxygen, culminating in the production of singlet oxygen species (1O2). (**D**) Damage to cellular macromolecules leads to tumor cell death by different processes, such as apoptotic, necrotic and autophagic mechanisms.

PDT has emerged as a promising alternative or as a possible combination to conventional treatments, such as surgery, chemotherapy and radiotherapy [12]. It is based on the use of a systemically or locally administrated photosensitive molecule. Once “excited” by light at an appropriate wavelength, the photosensitive molecule undergoes electronic transitions to higher energy states, making it reactive to several compounds in the surrounding environment [13–18]. Photosensitizers, as these photosensitive molecules are known, are excited in the presence of light. In the presence of molecular oxygen, this process, leads to a series of photochemical reactions that culminate in the generation of free radicals and singlet oxygens (highly reactive chemical species derived from molecular oxygen). These photochemical reactions cause oxidative damage and target cell death [13, 14]. The existence of modern laboratory apparatuses and fiber-optic endoscopy systems allow for light application at the appropriate wavelength in several parts of the body, permitting PDT application to internal tumors [19]. Figure 1 illustrates photodynamic therapy principles.

PDT efficiency depends on a high number of physicochemical and physiological properties, such as type of photosensitizer, drug concentrations, location of the photosensitizer in the tumor, adequate dosimetry (total light dose, exposure time and form of light exposure) and oxygen availability [16, 17]. PDT success requires specific conditions aiming at the high production of singlet oxygen, since numerous studies have demonstrated that this chemical species is the primary responsible for PDT cytotoxic effects [15, 20, 21]. Tissue light penetration is dependent on tissue characteristics and wavelength. Wavelengths in the red or infrared range are recommended for clinical application, since they exhibit greater tissue penetration within a “therapeutic window”, from 600 nm to 800 nm, which produces enough energy to support the formation of singlet oxygen species [15, 18]. To overcome this light penetration problem, Chen *et al.,* 2006 [22] proposed the use of synthesized nanoparticles (nanoscintillators), which can absorb X-rays and generate visible light.

### Nanoscintillators

Scintillation is the capability that certain substances present to emit light, triggered by interactions with ionizing radiation. Nanoparticles, as the name suggests, range from 10 to 1000 nm, although in terms of biological significance, the range from 10 to 100 nm is the most applied [23]. Nanoscintillators are composed of inorganic salts, most doped with rare earth metals as depicted in Table 1.

**Table 1:** Use of X- rays with nanoscintillators in different biological models

Year	Nanoparticle	Size	Nanoparticle concentration	Photosensitizer	X-rays energy	Biological Model	References
2008	LaF_3_:Tb^3+^	15 nm	0.035 M	Meso-tetra (4-carboxyphenyl) porphine (MTCP)	120 keV	N/A	Liu YF, *et al*. [88]
2010	ZnO nanorods (NRs)	0.5 μm	N/A	P rotoporphyrin dimethyl ester (PPDME)	N/A	T47D cells	Kishwar S, *et al*. [89]
2011	Y_2_O_3_	12 nm	2.5–95 μg/mL	Psoralen	2 Gy, 160 or 320 kVp	PC3 cells	Scaffidi JP, *et al*. [90]
2011	Gd_2_O_2_S:Tb	20 μm	5 mg/mL	Photofrin II	120 keV, 20 mAs	Human glioblastoma cells	Abliz E, *et al*. [91]
2013	Tb_2_O_3_	10 nm	1 mM	Porphyrin	N/A	N/A	Bulin AL, *et al*. [92]
2013	ZnO	50 nm	0.3–0.6 μM	meso-tetra (4-sulfonatophenyl) porphyrin (TSPP)	N/A	Escherichia coli	Senthilkumar S, *et al*. [93]
2014	LaF_3_:Ce^3+^	2 μm	1 μg/mL	Protoporphyrin IX (PPIX)	3 Gy	PC3 cells	Zou X, *et al*. [94]
2016	Sr_2_MgSi_2_O_7_:Eu^2+^, Dy^3+^	273 nm	10 μg/mL	Protoporphyrin IX (PPIX)	1-7 Gy	PC3	Homayoni H, *et al*. [95]
2014	Cu−Cy	50−100 nm	50 μg	Self	5 Gy	MCF-7 xenograft	L Ma, *et al*. [96]
2016	ZnS:Cu, Co	4 nm	0.05 mM	Tetrabromorhodamine-123 (TBrRh123)	2 Gy	PC3 cells	L Ma, *et al*. [97]
2015	SrAl_2_O_4_:Eu^2+^	80 nm	50 μg/mL	Merocyanine 540 (MC540)	0.5 Gy	U87MG xenograft	Chen H, *et al*. [98]
2015	LaF_3_:Tb	3−45 nm	N/A	Rose Bengal (RB)	2−10 keV	N/A	Tang Y, *et al*. [99]
2015	LaF_3_:Tb	3−45 nm	20 mg/mL	Rose Bengal (RB)	N/A	Tumor model	Elmenoufy AH, *et al*. [100]
2016	CeF_3_	7−11 nm	0.1–0.9 μM	Veterporfin (VP)	6 Gy, 8 keV, or 6 MeV	Panc-1	Clement S, *et al*. [101]
2015	LiYF4:Ce^3+^	34 nm	25-50 μg/mL	ZnO	8 Gy	HeLa cells	Zhang C, *et al*. [102]
2015	SiC/SiOx NWs	20 nm	50 μg/mL	Porphyrin	2 Gy, 6 MV	A549 cells	Rossi F, *et al*. [103]
2015	ZnO/SiO_3_	98 nm	0.005–0.05 M	ZnO	200 kVp, 2 Gy	LNCaP and Du145 cells	Generalov R, *et al*. [104]
2015	GdEuC_12_ micelle	4.6 nm	500 μM	Hypericin (Hyp)	5–40 KeV	HeLa cells	Kascakova S, *et al*. [105]

N/A = Not applicable.

The great advantage of nanoscintillators is the possibility of applying low energy X-rays (300 KeV) to generate light, thus diminishing radiotherapy side effects or allowing for combined use with chemotherapy. Radiotherapy normally applied energies ranging from 6 to 20 MeV. X-rays show great penetration capacity, reaching regions inaccessible to visible light, such as the brain or deep regions in tumors (more than 5 mm).

### Photodynamic therapy clinical applications

PDT is a promising alternative for the treatment of infectious diseases and infected blood and derivatives, as well as for the photoinactivation of multiresistant strains or microbial biofilms [24–26] (see Table 2). In comparison to the usual cancer treatment procedures (radiotherapy, chemotherapy and surgery), PDT displays a number of advantages. It is non-invasive and can be applied to virtually all types of cancer. For example, Temoporfin Foscan^®^ (a first generation photosensitizer), was applied for the treatment of lung, gastric, prostate and skin cancers [27]. PDT may also be used repeatedly without producing side effects, usually showing excellent healing results [28]. In addition, tissue preservation leads to practically no fibrosis, thereby conserving the functional anatomy and mechanical integrity of the organs undergoing the procedure, as well as selective tumor removal without secondary detrimental effect on surrounding healthy tissues [29]. PDT can also be used before or after aforementioned conventional treatments without compromising these therapeutic modalities. In addition, due to the inactivation of the photosensitizer in the absence of light, PTD shows low systemic toxicity. Finally, many PDT procedures can be performed in an ambulatory environment, lowering costs and making the procedure more acceptable to patients, resulting in increased patient adherence to treatment [28, 30, 31].

**Table 2:** Use of MB in different PDT

Year	Patient number	MB concentration	Administration	Indication	Light Energy	Wavelength light source	References
2005	60	2% dissolved in acetone	Injection	Onychomycosis	18 J/cm^2^	600 to 750 nm	Tardivo JP, *et al*. [46]
2005	10	2% aqueous solution	Intratumor injection	Metastatic melanoma, Basal cell carcinoma, Squamous cell, Breast cancer, Kaposi's sarcoma.	18 to 36 J/cm^2^	600 to 750 nm	Tardivo JP, *et al*. [46, 85]
2015	20	2% aqueous solution and 0,2% hydrogel	Intralesional injection and topically	Nodular or ulcerative basal cell carcinoma	48 J/cm^2^	N.I	Samy NA, *et al*. [106]
1997	3	10%	Topically	chronic plaque-stage psoriasis	5 J/cm^2^	600 to 700 nm	Schick E, *et al*. [107]
2006	26	5% aqueous solution	Gargle	Oral Lichen Planus	120 J/cm^2^	632 nm	Aghahosseini F, *et al*. [108]
2009	16	0,1% hydrogel	Topically	Resistant psoriatic plaque	565 mW	670 nm	Salah M, *et al*. [109]
2014	80	2% aqueous solution	Oral administration	onychomycosis	18 J/cm^2^	630 nm	Figueiredo Souza L W, *et al*. [110]
2009	13	0,1% hydrogel	Topically	Acne vulgaris			Fadel M, *et al*. [111]
2014	18	1% aqueous solution	Irrigation using syringes and catheters	Neuropathy, ulceration and infection in diabetic patin	30 J/cm^2^	400 to 725 nm	Tardivo JP, *et al*. [112]
1995	3	1% aqueous solution	Intratumor injection	Inoperable oesophageal tumours	7 J/cm^2^	662 nm	Orth K, *et al*. [52]
2012	12	0.01%	applied at the bottom of the periodontal pocket	Periodontitis in HIV patients	0,03 W	660 nm	Noro Filho, *et al*. [113]

N.I = not informed.

### Photosensitizers

Photosensitizing (PS) agents are essential PDT components. They are responsible for transferring the energy required for the occurrence of photochemical reactions that result in selective cell or tissue destruction [32]. A wide range of natural and synthetic compounds are capable of absorbing radiation in the UV-visible spectrum and generating singlet oxygen species, such as plant-derived products and synthetic macrocyclic complexes [14]. To function as a photosensitizer, a molecule must exhibit the following properties: (1) a high absorption coefficient in the spectral region of the excitation light source; (2) an appropriate triplet energy state (E_T_ ≥ 95 kJ.mol^-1^), in order to transfer to molecular oxygen in the ground state; (3) a high quantum triplet yield (φ_T_ ≥ 0.4), long triplet half-life state (τ_T_ ≥ 1 μs) and (4) a high photostability state [33].

Over 1450 molecules with potential PDT applications have been catalogued [34]. However, only some may be applied for this purpose, since the costs for their introduction into the clinical practice related to clinical tests are quite high. Thus, only sensitizers with “exceptional” properties justify the financial expenditure [34]. In addition to suitable chemical and physical properties, other characteristics are recommended for a drug to be considered optimal for PDT, including high chemical purity and rigorous and simple chemical synthesis; preferential accumulation at the site of interest; lack of toxicity in the dark; strong phototoxicity; rapid clearance from normal tissues; amphiphilicity; easy administration by various routes; low manufacturing cost and easy storage and marketing [15, 35, 36].

Photosensitizers can be classified according to their structure or origin. A traditional classification separates these compounds into three generations. Porphyrins and other substances developed in the 70's or early 80's are named first-generation photosensitizers, while porphyrin derivatives or synthetic compounds produced in the late 80's are termed second-generation photosensitizers. Third generation photosensitizers refer to changes carried out in their structures leading to selective tumor accumulation, like organic conjugates (liposomes or conjugated to antibodies) [32, 35]. Furthermore, existing photosensitizers can be classified into three large families: (1) porphyrin-based photosensitizers (eg, Photofrin, ALA/PpIX, BPD-MA); (2) chlorophyll-based photosensitizers (eg chlorins, purplish, bacteriochlorins); and (3) dyes (phthalocyanines, naphthalocyanines, methylene blue) [35].

Clinically- or experimentally-applied photosensitizers include many metallocomplexes which generally exhibit maximum absorption bands in the visible red spectrum and a molar extinction coefficient ranging from 10^3^ to 10^5^ M^−1^.cm^−1^ [37]. Most are based on a tetrapyrrole aromatic nucleus, similar to the protoporphyrin found in hemoglobin. These rings exhibit a relatively broad absorption band in the 400 nm region, known as the Soret band, and a group of minor bands directed to red wavelengths, known as the Q-band [38]. Hematoporphyrin derivatives (HpD) and porfirmer sodium (Photofrin) obtained from subsequent purifications are prototype drugs and were the first to be applied in the clinical practice. Despite their success as tools for the treatment of cancer and other conditions, these compounds display some disadvantages, such as lack of chemical homogeneity, slow skin clearance, resulting in long-term photosensitivity, and poor absorption in the clinical wavelength (ε = 10^3^ M^−1^. cm^−1^ at 640 nm), where light shows adequate tissue penetration [14, 36]. This has motivated the search for new photosensitizers that would exceed these limitations and present appropriate photophysical and photochemical properties.

### Methylene blue (MB) characteristics

Among second-generation photosensitizers, phenothiazine dyes are noted for displaying desirable photophysical and photochemical characteristics for PDT applications, such as high production of singlet oxygen species and strong absorption in therapeutic range (600–750 nm) [39]. Chemically, these compounds are formed by two main portions, namely chromospheres, which are aromatic ring systems with delocalized p-electrons, and peripheral modifications, such as side chains and auxochromes. This heterocyclic ring system has long been established and its chemical synthesis is well understood, allowing for easy analogue preparation [40]. Two main classes of phenothiazines are used in the medical practice: oxidized phenothiazines, planar and tricyclic (MB, thionine and toluidine blue), and non-oxidized molecules, such as promethazine and chlorpromazine, that are not completely aromatic or planar. Typically, phenothiazine dyes present a delocalized positive charge at neutral pH in the ring system, whereas phenothiazine neuroleptics exhibit a positive charge located at the nitrogen distal side chain. Phenothiazine compounds display great versatility concerning possible applications in medical practice, and are studied regarding their therapeutic role in the local treatment of bacterial infections, tuberculosis, trypanosomiasis, malaria, rickettsia infections, fungal infections and cancer [41]. Regarding cancer PDT applications, the most researched compounds are methylene blue (IUPAC name: 3,7-bis(Dimethylamino)-phenothiazin-5-ium chloride) and toluidine blue, which are structurally and physicochemically similar. Both possess a nitrogen atom capable of accepting protons in the center ring and two nitrogen atoms that contribute to charge relocation in the chromophore by stabilizing the cationic form.

MB has been widely applied as a vital dye for over a century [42, 43]. It was first synthesized in the nineteenth century (1876) by Heinrich Caro, who worked at Badische Anilin und Soda-Fabrik, a German chemical industry, from the oxidation of p-dimethylaniline [44]. In the late nineteenth century, scientists Robert Koch and Paul Ehrlich used MB to stain microorganisms. Based on these studies, Erlich envisioned that these could also be applied therapeutically. In fact, in 1891, he demonstrated that MB was effective in treating malaria in humans [45].

MB shows an intense color in aqueous solutions due to the intense absorption of the phenothiazine chromophore at 600–700 nm in the visible spectrum, with a well-defined peak at 664–666 nm [39, 46]. A less intense peak can be observed in or near the UV region, particularly from 284 to 300 nm [42]. Moreover, this compound can also generate radicals, even in the presence of reducing agents [46]. In aqueous solutions, the excitation spectrum is concentration-dependent due to dimerization and oligomerization phenomena, whose equilibrium constant is 3.8 × 10^−3^ M^−1^ [46]. Between 10^−5^ and 10^−3^ M, MB aggregation is limited to dimer formation. Dimerization increases with ionic strength and can increase or decrease the presence of charged interfaces, depending on the relationship between the dye and the interface [47]. Monomers and dimers have very distinct absorption bands, with the dimer band 60 nm shorter compared to the monomer band. Similarly to many inorganic dyes, MB does not follow the Beer-Lambert law [43], probably due to the reversible formation of polymers, which are maintained together by the dispersion forces originating from delocalized p electrons in the individual dye molecules [44]. The photodynamic mechanism for MB is quite complex. It has a high quantum yield of intersystem crossing (Φ_Δ_ ~ 0.5), which can generate high singlet oxygen species concentrations and mediate cytotoxicity to form hydroxyl radicals, which in turn can alter intracellular Ca^2+^ homeostasis [48, 49].

MB is characterized by its low toxicity and can be used in the intraoperative or endoscopic marking of various tumors, as well as in the clinical treatment for methemoglobinemia [14, 49]. Although idiosyncratic reactions may occur, MB doses are high without the occurrence of measurable toxicity. For example, concentrations routinely used for staining the oral and nasopharyngeal mucosa are in the millimole range (1% w/w or 31,2 mM) [39]. Additionally, MB pharmacokinetics are well established and have shown different distribution profiles, depending on the administration route [50].

Several studies considering MB for the treatment of neoplasms have been reported. *In vitro* studies have demonstrated that MB shows phototoxicity against several tumor cell lines, such as cervical cancer adenocarcinomas (HeLa) and bladder carcinomas [51]. Local MB administration has also been applied in the treatment of inoperable esophageal tumor [52]. However, oncology MB clinical applications have been limited due to its lack of activity when applied systemically. This weak pharmaceutical activity results in poor penetration of tumor cellular environment [53].

### Strategies to improve MB cell entry

**Figure 2:**
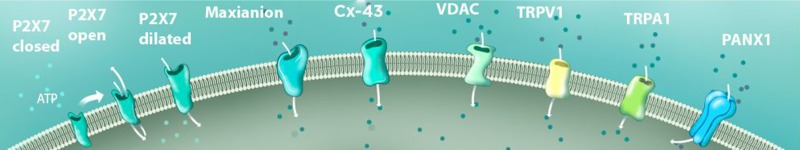
Using the pore associated with P2×7 receptor and other pores as an entry pathway for methylene blue (319 Da) in PDT

In this scenario, it is, thus, essential to search for pathways that may facilitate MB a hydrophilic drug, uptake into target cells, leading to feasible tumor treatments. In this context, pore-forming proteins are present in the plasma membrane of many mammalian cell types, able to open under physiological or pathophysiological conditions, such as changes in cell volume, hypoxia and alterations in extracellular pH, among others. When open, they allow for the passage of molecules of up to 1,000 Da without necessarily leading to cell death. Among pore-forming proteins, pannexin-1 [54], connexin hemichannels [55], TRPV1-4 subtypes and TRPA1 [56], calcium homeostasis modulator 1 (CALHM1) [57], Maxi anion [58], plasma membrane VDAC [59] and ATP-gated subtypes P2×2 [60–62], P2×4 [63–65] and, especially, P2×7 receptors [66–68] are noteworthy (Figure 2). One possibility would be to study the involvement of the P2×7 associated-pore as an MB uptake route in the cytoplasm of neoplastic cells, and, in this regard, we have recently demonstrated that P2×7 can function as a drug delivery system in the J774 tumor cell line [69].

Since this receptor is related to several physiological processes, such as T-cell maturation, innate immune response activation, epithelial secretion, mineralization regulation, bone resorption and fast synaptic transmission, among others, it is not surprising that it is also associated to several pathological conditions. Indeed, P2×7 polymorphisms have been associated with susceptibility to infectious neurodegenerative diseases, depression, osteoporosis and inflammatory diseases [70–77].

In this context, several groups have investigated the role of P2×7 in tumor pathophysiology, mainly due to its cytotoxic potential, which can exploited as a pharmacological target. Its expression (mRNA and protein) has been demonstrated in different tumor and tumor cell lines subtype (Table 3), but no consensus ion its function has been reached. Nevertheless, evidence clearly shows a dependence on the cellular model under study, as this receptor may be involved with growth/proliferation or death induction (apoptosis/necrosis) [78–84].

**Table 3:** P2×7 expression and functions in neoplasms

Year	Tumor type	Expression level	Physiological effect of the activation	References
2005	Prostate	Protein	No determined	Slater M, *et al*. [114]
2013	Breast	mRNA/Protein	Increases intracellular Ca^2+^ concentration	Jelassi B, *et al*. [115]
2008	Thyroid	mRNA/Protein	Not determined	Solini A, *et al.* [116]
2007	Pancreas	mRNA	Not determined	Kunzli BM, *et al.* [117]
2005	Skin carcinoma	mRNA	Not determined	Slater M, *et al.* [114]
2006	Uterine epithelial	mRNA/Protein	Not determined	Li X, *et al.* [118]
2006	Neuroblastoma	mRNA/Protein	Induce cell proliferation	Raffaghello L, *et al.* [119]
2005	Melanoma	mRNA/Protein	YO-PRO-1 uptake	White N, *et al.* [120]
2002	Chronic Lymphocytic Leukemia	mRNA/Protein	Decrease the proliferation	Adinolfi E, *et al.* [121]
2015	Hepatocellular carcinoma	mRNA/Protein	Not determined	Liu H, *et al.* [122]
2014	Ovarian carcinoma	mRNA/Protein	Increase of intracellular Ca^2+^ concentration, but not cell death	Vázquez-Cuevas FG, *et al.* [123]
2010	Papillary thyroid carcinoma	Protein	Not determined	Gu LQ, *et al.* [124]
2012	Lung cancer	mRNA/Protein	Ethidium uptake	Takai E, *et al.* [125]
2012	Glioma	mRNA/Protein	Ethidium uptake	Gehring MP, *et al.* [126]
2013	Colorectal cancer	mRNA	Ethidium uptake	Bian S, *et al.* [127]

### MB and the P2×7 receptor

**Figure 3:**
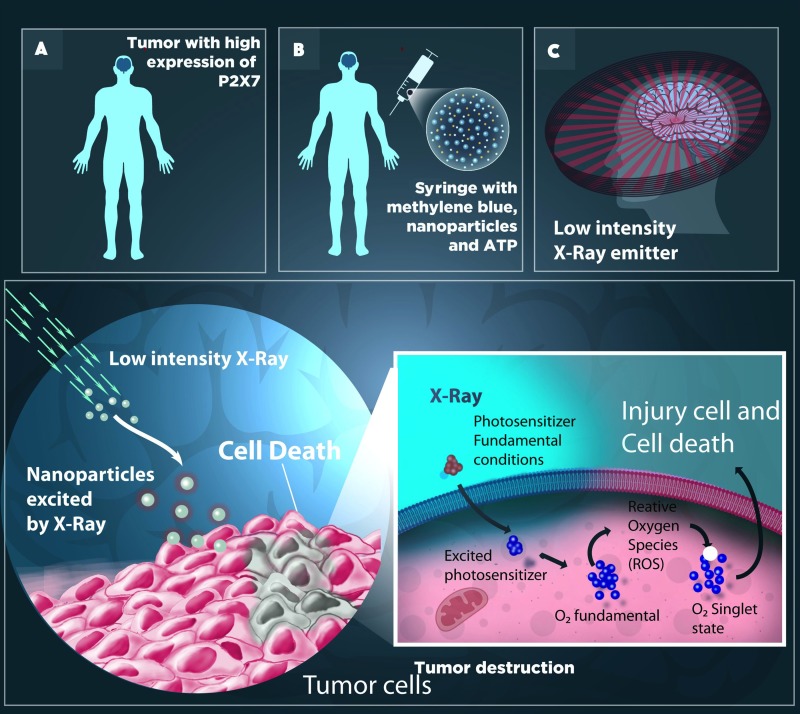
Photodynamic therapy may increase selectivity when applied concurrently to photosensitizing (PS) radioluminescent molecules (RL) (**A**) A tumor with high P2×7 expression can be treated with the application of Methylene blue (MB), a potent Photosensitizer (PS), nanoparticles and ATP. (**B**) These compounds in solution form are administered directly into the venous circulation via syringe and, once in the blood, can migrate to any part of the body. ATP administration activates the pore associated to the P2×7 receptor, allowing for MB passage. (**C**) The RL, once excited by low intensity X-rays, emit luminescence in the red spectrum. Thus, the luminescence produced by the radioluminescent molecule leads to an excited state of the photosensitizer (PS^*^), initiating a series of photochemical reactions in the tumor environment, resulting in the production of singlet oxygen species (1O2) from molecular oxygen (3O2), which is a highly reactive and cytotoxic chemical species.

Although knowledge on tumor biology has advanced greatly in recent years, the approval of new cancer treatment drugs is still limited. In addition, traditional therapies, such as chemotherapy and radiotherapy, lack selectivity and possess numerous side effects. Accordingly, PDT emerges as a promising alternative, displaying very positive results over the years. MB stands out among photosensitizers with possible applications in clinical practice, since it displays excellent photodynamic properties and low toxicity in humans. However, its use has been limited due to its low tumor penetration [85, 86] The literature describes several strategies to increase MB uptake by tumor tissues, such as altering its chemical structure in order to increase lipophilicity, or its association with three-dimensional structures, allowing access to the cytoplasm, as cited previously. Herein, we propose another possibility, of using transient membrane pores, such as the pore induced by activation of the P2×7 receptor. Interestingly, this receptor has been described in certain types of tumors, making it an adequate target for pharmacological tumor therapy. This strategy could enhance PDT once MB is at a size able to pass through the pore (319 Da). Figure 3 displays a scheme representing this hypothesis, in which a cell is exposed to MB along with ATP administration at concentrations sufficient enough to open the P2×7 pore for 15 to 20 minutes. After the incubation period, the cells would be exposed to light in the appropriate wavelength (600–700 nm) in order to activate MB photodynamic activity. Furthermore, following the system upgrades as displayed in Figure 3, radioluminescent molecules, which produce luminescence in the red spectrum excited by low-intensity X-ray, would become even more selective and exhibit fewer side effects [87], as previously outlined in Figure 3.
